# Effect of Auxin (IAA) on the Fast Vacuolar (FV) Channels in Red Beet (*Beta vulgaris* L.) Taproot Vacuoles

**DOI:** 10.3390/ijms21144876

**Published:** 2020-07-10

**Authors:** Zbigniew Burdach, Agnieszka Siemieniuk, Waldemar Karcz

**Affiliations:** Faculty of Natural Sciences, Institute of Biology, Biotechnology and Environmental Protection, University of Silesia in Katowice, 40-032 Katowice, Poland; zbigniew.burdach@us.edu.pl (Z.B.); agnieszka.siemieniuk@us.edu.pl (A.S.)

**Keywords:** *Beta vulgaris* L., FV channels, IAA, patch-clamp, vacuole

## Abstract

In contrast to the well-studied effect of auxin on the plasma membrane K^+^ channel activity, little is known about the role of this hormone in regulating the vacuolar K^+^ channels. Here, the patch-clamp technique was used to investigate the effect of auxin (IAA) on the fast-activating vacuolar (FV) channels. It was found that the macroscopic currents displayed instantaneous currents, which at the positive potentials were about three-fold greater compared to the one at the negative potentials. When auxin was added to the bath solution at a final concentration of 1 µM, it increased the outward currents by about 60%, but did not change the inward currents. The imposition of a ten-fold vacuole-to-cytosol KCl gradient stimulated the efflux of K^+^ from the vacuole into the cytosol and reduced the K^+^ current in the opposite direction. The addition of IAA to the bath solution with the 10/100 KCl gradient decreased the outward current and increased the inward current. Luminal auxin reduced both the outward and inward current by approximately 25% compared to the control. The single channel recordings demonstrated that cytosolic auxin changed the open probability of the FV channels at the positive voltages to a moderate extent, while it significantly increased the amplitudes of the single channel outward currents and the number of open channels. At the positive voltages, auxin did not change the unitary conductance of the single channels. We suggest that auxin regulates the activity of the fast-activating vacuolar (FV) channels, thereby causing changes of the K^+^ fluxes across the vacuolar membrane. This mechanism might serve to tightly adjust the volume of the vacuole during plant cell expansion.

## 1. Introduction

The plant vacuole is a dynamic cellular compartment that can occupy more than 90% of the cell volume and that is essential to plant growth and development. The increase in volume of plant cells during growth mainly occurs through an increase in the volume of the vacuolar sap, which usually is about an one order of magnitude larger compared to the volume of the cytosol [1]. It is well established that auxin is a crucial regulator of cell-volume control in plants. Recently, several reviews on the function, transport mechanisms and molecular composition of plant vacuoles have been published [2,3,4,5,6]. However, as was indicated from the studies cited in these reviews, our knowledge about the effect of auxin (indole-3-acetic acid, IAA) on the vacuolar ion transport is very limited, and much work is needed to answer the question of whether auxin is involved in regulating ion transport across the tonoplast. This question is very important taking into account the fact that auxin and its metabolites have been found in plant vacuoles, and that auxin transport across the tonoplast plays an essential role in maintaining auxin homeostasis [7].

In contrast to the vacuolar membrane, regulation of the plasma membrane ion transport by auxin (especially across K^+^ channels) is well documented in the literature [8,9,10,11,12]. It was found, at least in the *Vicia faba* guard cells and *Zea mays* coleoptile cells, that the K^+^ channels are activated by auxin. For example, Blatt and Thiel [8] showed that the currents through both the K^+^-inward and outward rectifiers from the *Vicia faba* guard cells are mediated by auxin (IAA), which is consistent with the ability of the hormone to promote stomatal opening or to inhibit it. Auxin had a bimodal effect on the K^+^-inward rectifier; at low concentrations (<10 µM), auxin stimulated the K^+^ conductance, whereas at high concentrations (>30 µM), it inhibited it. An additional line of evidence for the auxin-mediated control of the K^+^ channels relates to the electrophysiological experiments that were performed with maize coleoptile cells, which contain a typical plant K^+^-inward rectifier in the plasma membrane [9,11,12,13,14]. In these experiments, it was shown that auxin not only stimulated the K^+^-inward rectifier (ZMK1, *Zea mays* K^+^ channels) but also controlled its expression [9]. Both the guard and coleoptile cells mediate the K^+^ uptake in the course of cell expansion [9,12,15,16].

Three types of K^+^-permeable channels were found in the vacuolar membrane: the slow-activating (SV), fast-activating (FV) and K^+^-selective vacuolar (VK) channels (reviewed in [17]). SV channels are permeable to monovalent and divalent cations and are activated by the cytosol-positive voltages at an elevated cytosolic Ca^2+^ [18,19,20]. In turn, FV channels are permeable for only monovalent cations and mediate K^+^ at very low concentrations of cytosolic Ca^2+^ and large voltages of either polarity [18,21]. Moreover, the FV channels are inhibited by the divalent cations from either side of the vacuolar membrane [22,23,24]. A third type of vacuolar K^+^ (VK) channels is the Ca^2+^-activated channels, which are highly K^+^-selective channels [19].

We began to explore the vacuolar K^+^ channels of red beet taproots suspecting that these channels, similar to the plasma membrane K^+^ channels, are sensitive to auxin. Recently, we showed that auxin (IAA) stimulates the activity of the slow vacuolar (SV) channels in red beet taproot vacuoles [25]. However, in the present study, we addressed the question of whether IAA modulates the activity of the FV channels, which, in contrast to the SV channels, have a significant probability of being open in intact resting cells, i.e., at a low cytosolic Ca^2+^ concentration and voltages that are close to the resting tonoplast potential [22]. In addition, virtually no studies exist on the effect of IAA on the activity of the FV channels. From the patch-clamp experiments described here, we suggest that auxin regulates the activity of the fast-activating vacuolar (FV) channels, thereby causing changes of the K^+^ fluxes across the vacuolar membrane.

## 2. Results

### 2.1. Cytosolic Auxin at 1 µM Stimulated the Instantaneous Outward Current of the Whole-Vacuole FV Channels

**Figure 1:**
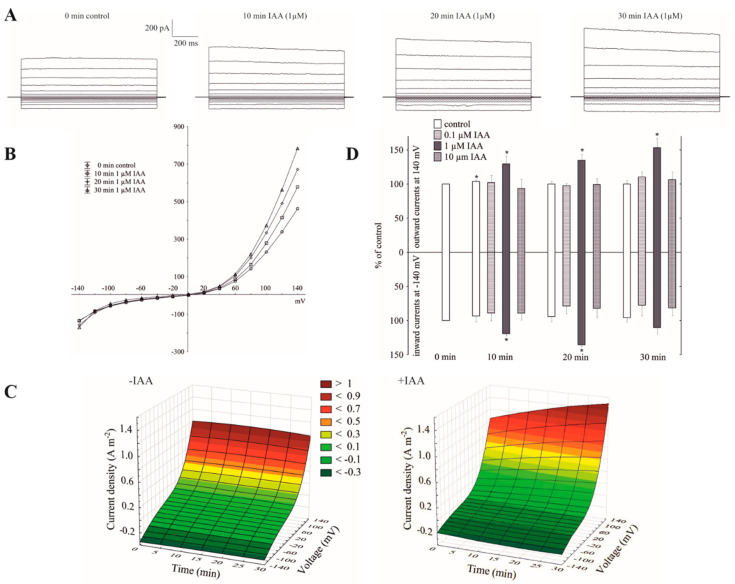
Effect of cytosolic auxin (indole-3-acetic acid, IAA) on the fast-activating currents in red beet taproot vacuoles. (**A**), An example of a whole-vacuole fast-activating vacuolar (FV) current recording in the control bath solution and in the presence of 1 µM IAA. The control at “0 time” (0 min control) means the whole-vacuole FV current that was recorded in a symmetrical 100 mM KCl immediately after gaining access to the whole vacuole; however, the current in the absence and presence of IAA was recorded 5, 10, 15, 20, 25 and 30 min later. For clarity, only the FV currents in the control (0 min control) and the presence of IAA after 10, 20 and 30 min are shown. (**B**), The instantaneous vacuolar currents shown as a function of voltage (the data are from the same experiment as shown in A). (**C**), The current–voltage relationships in the absence (−IAA) and presence (+IAA) of 1 µM IAA shown as a function of time. The means of at least seven different vacuoles are shown. (**D**), The dependence of the whole-vacuole FV currents (recorded at −140 and +140 mV) on the cytosolic IAA concentration and time. The data are expressed as a % of the control (0 min control). The means ± SE of at least seven different vacuoles are shown. The normal distribution was evaluated by the Shapiro–Wilk test (*p* > 0.05). The statistical differences between control at 0 min and other variants at the same voltage were analyzed using a paired *t*-test (*p* < 0.05). Asterisk (*) indicates significant difference.

Patch-clamp recordings performed in experimental conditions described here, e.g., 100 mM KCl on both sides of the tonoplast, low Ca^2+^ (<10 nM) at the cytosolic side and neither added nor buffered Ca^2+^ at the vacuolar side (see also Materials and Methods), revealed typical FV channel currents in the red beet (*Beta vulgaris* L.) taproot vacuoles (Figure 1A–C). The whole vacuolar currents recorded in these conditions displayed instantaneous currents, which at positive potentials (outward current) were about three-fold greater compared to the one at the negative potentials (inward current). When auxin was added to the bath solution at a final concentration of 1 µM, it caused an increase in the outward currents by about 60%, whereas it did not change the inward currents over 30 min at ±140 mV, respectively, compared to the control (0 min control; Figure 1A–D). The current–voltage relationships, which are shown as a function of time, indicate that in the presence of 1 µM IAA, the amplitudes of the whole-vacuole currents increased proportionally at the positive potentials compared to the control (Figure 1C). Auxin at 0.1 and 10 µM did not cause any significant changes in the amplitude of the instantaneous currents over 30 min at ±140 mV (Figure 1D). Moreover, as can be seen in Figure 1D, there was only a small rundown of the FV currents (<10%) at positive potentials.

### 2.2. Cytosolic Auxin at Vacuole-to-Cytosol KCl Gradient Predominantly Increased the K^+^ Efflux from the Vacuole into the Cytoplasm

**Figure 2:**
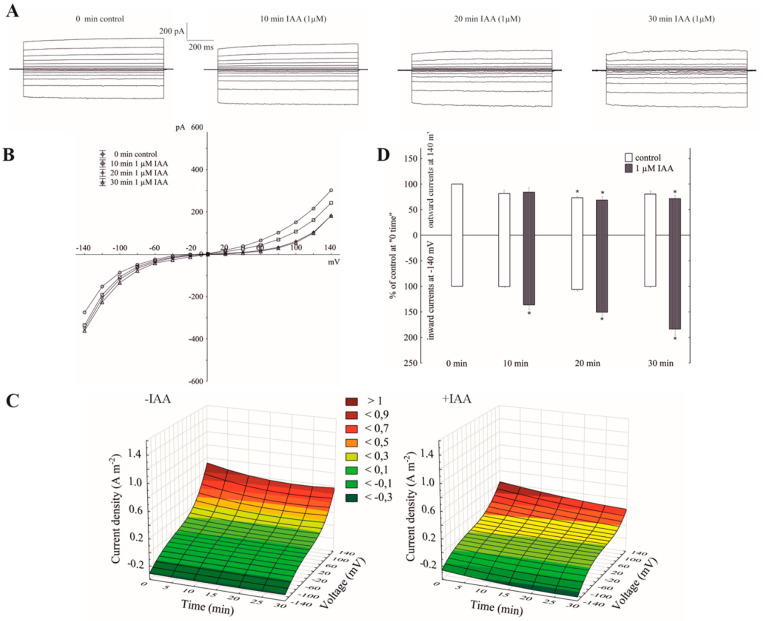
The amplitudes of the instantaneous vacuolar currents in the presence of cytosolic IAA and a ten-fold vacuole-to-cytosol KCl gradient. (**A**), An example of a whole-vacuole FV current recording in a 10/100 KCl gradient in the absence and presence of 1 µM IAA. The control at “0 time” (0 min control) means the whole-vacuole FV current was recorded immediately after establishing the whole-vacuole configuration in the ten-fold KCl gradient that was imposed on a vacuolar membrane; however, the current in the absence and presence of IAA was recorded 5, 10, 15, 20, 25 and 30 min later. For clarity, only the FV currents in the control (0 min control) and the presence of IAA after 10, 20 and 30 min were shown. (**B**), The instantaneous vacuolar currents shown as a function of voltage (the data are from the same experiment as shown in A). (**C**), The current–voltage relationships in the absence (−IAA) and presence (+IAA) of 1 µM IAA shown as a function of time. Means of at least seven different vacuoles are shown. (**D**), The whole-vacuole FV currents recorded at −140 and +140 mV in the presence of 1 µM IAA. The data were expressed as % of the control (0 min control). Means ± SE of at least six different vacuoles are shown. The normal distribution was evaluated by the Shapiro–Wilk test (*p* > 0.05). The statistical differences between the control at 0 min and other variants at the same voltage were analyzed using a paired *t*-test (*p* < 0.05). Asterisk (*) indicates significant difference.

The imposition of a ten-fold KCl gradient on a vacuolar membrane (100 mM KCl in the bath solution was substituted with 10 mM KCl) resulted in about a 1.5-fold decrease of the outward current and no significant changes of the inward currents compared to the recordings in symmetrical 100 mM KCl, respectively (see the control in Figure 1A,C, and Figure 2A,C). It can also be seen from the data in Figure 2B that a decrease of cytosolic K^+^ from 100 to 10 mM caused a gradual positive shift (by about 40 mV) of the rise of the outward current, while the inward current was unchanged. The obtained results mean that in symmetrical 100 mM KCl at positive potentials (relative to the cytoplasm) an efflux of the K^+^ from the cytoplasm into the vacuole predominantly occurs, whereas the imposition of a ten-fold KCl gradient on a vacuolar membrane significantly reduced it. At the negative potentials, which, in turn, means that the K^+^ predominantly flow from the vacuole to the cytoplasm, the imposition of the KCl gradient did not cause any significant changes in the K^+^ efflux from the vacuole into the cytosol. However, when IAA at 1 µM was added to the bath solution at the 10/100 KCl gradient, it decreased the outward current by about 30% and increased the inward current by about two-fold compared to the control over 30 min at ±140, respectively (Figure 2A,B,D). This means that when a ten-fold KCl gradient was imposed on a vacuolar membrane, auxin caused both a decrease of the K^+^ efflux from the cytoplasm into the vacuole and an increase of the K^+^ efflux from the vacuole into the cytoplasm.

### 2.3. Vacuolar Auxin Diminished Whole-Vacuole FV Currents

**Figure 3:**
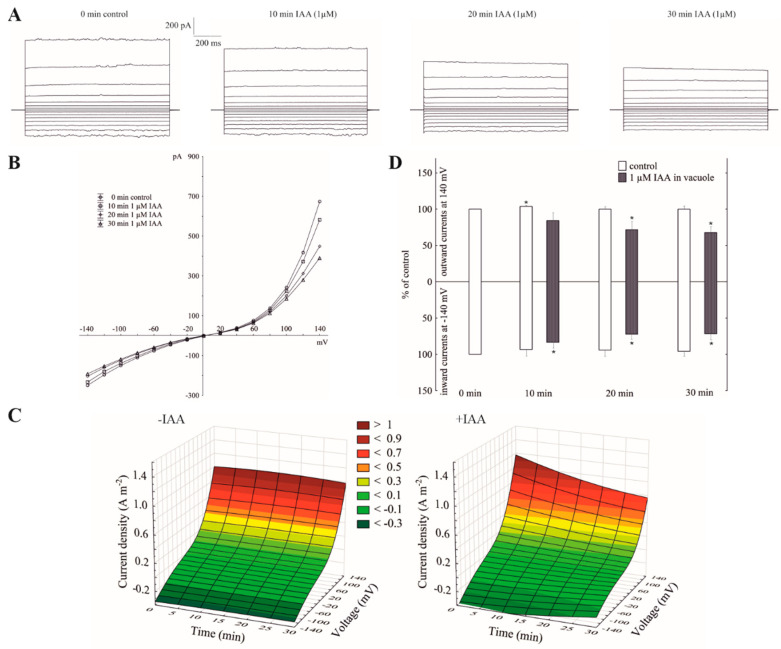
Effect of vacuolar auxin (1 µM IAA) on the fast-activating currents. (**A**), An example of a whole-vacuole FV current recording in symmetrical 100 mM KCl and in the presence of vacuolar IAA (auxin was added to the pipette solution, luminal IAA). Control at “0 time” (0 min control) means the whole-vacuole FV current was recorded immediately after gaining the access to the whole vacuole in the control medium; however, the current in the absence and presence of IAA was recorded 5, 10, 15, 20, 25 and 30 min later. For clarity, only the FV currents in the control (0 min control) and presence of IAA after 10, 20 and 30 min were shown. (**B**), The instantaneous vacuolar currents shown as a function of voltage (the data are from the same experiment as shown in A). (**C**), The current–voltage relationships shown as a function of time. The whole-vacuole currents were recorded in symmetrical 100 mM KCl in the absence (−IAA) and presence (+IAA) of vacuolar IAA. The data are the means of at least six independent experiments. (**D**), The whole-vacuole FV currents recorded at −140 and +140 mV in the presence of 1 µM IAA in a vacuole. The data are expressed as a % of the control (0 min control). The means ± SE of at least six different vacuoles are shown. The normal distribution was evaluated by the Shapiro–Wilk test (*p* > 0.05). The statistical differences between control at 0 min and other variants at the same voltage were analyzed a using paired *t*-test (*p* < 0.05). Asterisk (*) indicates significant difference.

When the whole-vacuole FV currents were recorded in symmetrical 100 mM KCl and in the presence of vacuolar auxin (luminal IAA at 1 µM), the FV currents decreased in a voltage- and time-dependent manner (Figure 3A–C). Luminal auxin diminished both the outward and inward current by about 25% compared to the control over 30 min at ±140 mV (Figure 3D). By comparing the current–voltage relationships shown as a function of time in symmetrical 100 mM KCl (Figure 1C) with ones at vacuolar auxin (Figure 3C), it should be stated that the control (0 min control) in the first case (cytosolic auxin) was lower than in the second case (vacuolar auxin), respectively.

### 2.4. Cytosolic Auxin Modulates the FV Single-Channel Currents in Symmetrical 100 mM KCl

**Figure 4:**
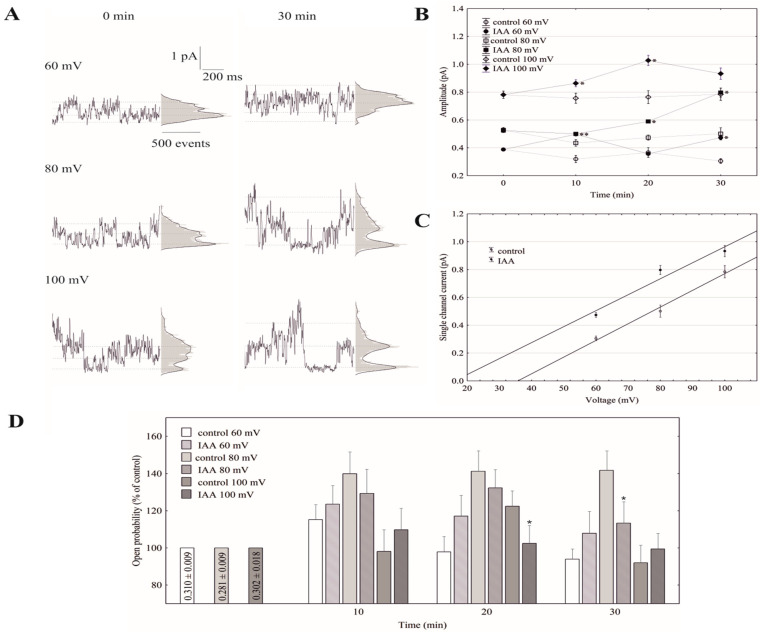
Effect of auxin on the single-channel activity in red beet taproot vacuoles. (**A**), The representative single-channel activity of the outside-out patch in symmetrical 100 mM KCl and in the absence (−IAA) and presence (+IAA) of auxin at 1 µM is shown. The current traces after 30 min are shown at the different membrane voltages that are indicated as adjacent to the traces. The dotted lines indicate the closing state and different opening ones. In this patch, there are at least three channels opening. The corresponding amplitude histograms are shown to the right of the current traces. (**B**), The current–voltage relationships of the FV single-channel current amplitudes that were obtained with and without auxin as a function of time. The values of the current were obtained as the differences of the maximum of the current histograms, and represent the open and closed states of a channel, respectively. The data points are the means (±SE) from at least six independent experiments. The data failed normal distribution (Shapiro–Wilk test, *p* > 0.05) hence, statistical differences between IAA and the control in the same time and voltage were tested using the non-parametric Mann–Whitney *U*-test (asymptotic significance two-tailed, *p* < 0.05). Asterisk (*) indicates a significant difference. (**C**), The current–voltage characteristics for single FV channels in the control bath and in the presence of IAA. Points were obtained from the experiments shown at 30 min in (**B**). As can be seen, IAA did not change the slope of simple linear regression, expressing the single channel conductivity. (**D**), The open probability of the FV single channels (expressed as % of control) is shown as a function of voltage and time. The open probability was calculated (using FitMaster software, see also [26]) as the sum of the channel open times in the current traces normalized to the total time of the traces divided by the number of active channels in the patch. The data points are the means (±SE) from seven independent experiments. The normal distribution was confirmed by the Shapiro–Wilk test (*p* > 0.05). The statistical differences of open probability values between control and IAA variants in the same time and voltage were analyzed using independent samples *t*-Test (*p* < 0.05). Asterisk (*) indicates significant difference.

Under conditions that enabled the observation of the whole-vacuole currents, the single-channel activities of the cytosolic side-out patches were also recorded. Auxin at a final concentration of 1 µM was added to the bath solution after obtaining a stable patch. Since in symmetrical 100 mM KCl, auxin only significantly changed the whole-vacuole outward current, we decided to perform further studies with single channels at positive voltages. Figure 4A shows the fast-activating currents in excised cytosolic side-out patches that had been bathed in the absence and presence of cytosolic IAA. As can be seen in Figure 4A, adding auxin to the bath solution resulted in a progressive increase of single-channel current amplitudes with increasing voltages (60, 80 and 100 mV) over 30 min compared to the control. In the presence of auxin, the amplitude of the fast-activating single channel currents generally increased with increasing voltage and time compared to the control (Figure 4B). The recordings of the single channel activity, which were obtained with and without auxin, showed that auxin did not change the unitary conductance of the single channels. The average single channel conductance in the presence of IAA was 12.83 ± 0.76 pS, which was not significantly different from the control (12.50 ± 0.47 pS; Figure 4C). The data from five patches that were performed in the outside-out configuration showed moderate differences in the open probability of single channels in the variants with and without auxin (Figure 4D). Considering the average number of open FV channels per µm^2^ of the vacuolar membrane, which was calculated as the open probability of a single channel multiplied by the number of channels per µm^2^, it should be stated that auxin increased the number of open channels by about 47% compared to the control (195 channels per 1000 per µm^2^) after 30 min at 100 mV. Since changes of the macroscopic currents depend on either changes of the single channel amplitudes or changes in the mean number of open channels or a combination of both parameters, it should be noted that auxin increased the macroscopic currents via a mechanism that involved both parameters. Thus, the voltage-dependent single-channel activity of the cytosolic side-out patches was consistent with the studies under the whole-vacuole configuration in which there was an increase of the outward current in the presence of auxin.

## 3. Discussion

It is well established that plant cells accumulate large amounts of K^+^ ions in their vacuoles, which occupy most of the intracellular volume of cells and are the main cellular reservoir for K^+^. Vacuolar K^+^ plays a key role, among others, in regulating osmotic/turgor and cell expansion (recently reviewed in Ragel et al., [27]). The vacuolar K^+^ channels, the predominantly SV and FV channels, control the K^+^ distribution between the vacuole and the cytosol [28]. Their activity is regulated by the cytosolic free Ca^2+^ concentrations (reviewed in [17,29]). At low cytosolic free Ca^2+^, FV channels constitute the main pathway for the flow of the K^+^ ions through the tonoplast. It is currently believed that the physiological roles of the FV channels are connected with maintaining the cellular K^+^ homeostasis [30], thereby providing a shunt conductance for the V-ATPase and the regulation of the tonoplast potential [22,31] and to a recently proposed role of the FV channels for conferring salinity tolerance in quinoa [32,33].

Little is known about the effect of auxin on the activity of the vacuolar K^+^ channels. To the best of our knowledge, this is the first report concerning the effect of IAA on the fast-activating vacuolar (FV) channels. Recently, we showed [25] that in symmetrical 100 mM KCl, when auxin was added to the bath solution at a final concentration of 1 µM, it stimulated the slow-activating vacuolar (SV) channels in red beet taproot vacuoles. This effect resulted from faster channel activation and an increased amplitude and in the number of opened SV channels. Interestingly, the addition of IAA to the bath solution with the same composition as the one that was used in the patch-clamp experiments caused a decrease of the vacuole volume [25]. However, the results described here showed that in the experimental conditions that were used for our recordings, e.g., 100 mM KCl on both sides of the tonoplast, low Ca^2+^ on the cytosolic side but no added and no buffered Ca^2+^ on the vacuolar side, the macroscopic vacuolar currents displayed an instantaneous currents, which at the positive potentials were about three-fold greater compared to the one at the negative potentials. Moreover, at the potentials in the range −20 and −60 mV, the activity of the FV channels was minimal. These findings are characteristic for the FV channel-mediated currents and were previously described by others [17,18,21,22,29]. When auxin was added to the bath solution at a final concentration of 1 µM, it increased the instantaneous outward currents by about 60%, but it did not change the inward currents (Figure 1). This observation suggests that in symmetrical 100 mM KCl, when auxin was added to the bath solution, it increased the K^+^ efflux from the cytoplasm into the vacuole. At 0.1 and 10 µM, auxin did not cause any significant changes of the outward currents. In turn, the imposition of the ten-fold vacuole-to-cytosol KCl gradient caused both a significant (by about 100%) increase of the K^+^ efflux from the vacuole into the cytosol (inward current) and a decrease (by about 30%) of the K^+^ current in the opposite direction (outward current) (Figure 2). Qualitatively similar changes, concerning a ten-fold KCl gradient, which was imposed on the vacuolar membrane, were also observed by others [21,22]. A comparison of Figure 1B and Figure 2B shows that the decrease of cytosolic K^+^ from 100 to 10 mM caused a positive voltage shift of the FV current voltage dependence to the right, i.e., the outward current became activated at the more positive potentials. However, when IAA at 1 µM was added to the bath solution at the 10/100 KCl gradient it decreased the outward current and increased the inward current compared to the control (Figure 2). Moreover, it was also shown that in symmetrical KCl, luminal auxin reduced both the outward and inward currents by about 25% compared to the control over 30 min at ±140 mV (Figure 3). The single channels, which were recorded in symmetrical KCl, demonstrated that cytosolic auxin changed the open probability of the FV channels at the positive voltages to a moderate extent, while it significantly increased the amplitude of the single channel outward currents as well as the number of open channels (Figure 4). At positive voltages, auxin did not change the unitary conductance of the single channels. While the present data do not explain the mechanisms by which auxin regulates the activity of the FV channels in red beet taproot vacuoles, it shows that auxin increases the amplitude of the single channel outward currents and the number of open channels in the vacuolar membrane, in the latter case, probably as a result of the unblocking of the “silent” channels. Both possibilities could be due to either an interaction of the IAA anions (which dominate at the pH of the bath solution, pH 7.5) with channel gating or an interaction of auxin with the lipids of the lipid-bilayer. The impact of auxin on the lipid-bilayer was recently reported by Hąc-Wydro et al. [34]. It should also be presumed that at low cytosolic Ca^2+^ concentrations and with no buffered Ca^2+^ of the vacuolar sap, the FV channels might play the role of the SV channels. However, taking into account the fact that under physiological conditions the FV channels predominantly mediate the K^+^ uptake to the vacuole, they are similar to the plasma membrane inward rectifying K^+^ channels (mentioned in the Introduction), the more so because both channels are regulated by auxin. The results presented here suggest that auxin regulates the activity of the fast-activating vacuolar (FV) channels, thereby causing changes of the K^+^ fluxes across the vacuolar membrane. This mechanism is probably involved in adjusting the volume of the vacuole during plant cell expansion. Taking the above into account, it might be hypothesized that auxin-mediated changes in FV channels activity are the key player in the regulating of vacuolar volume.

## 4. Materials and Methods

Red beet (*Beta vulgaris* L.) taproot vacuoles were isolated using the nonenzymatic method that was previously described by Coyaud et al. [35]. In accordance with this method, the vacuoles were directly extruded into a recording chamber by cutting a slice of fresh tissue and rinsing the surface with a bathing solution. The control bath solution in the patch-clamp experiments contained 100 mM KCl, 0.5 mM EDTA, 5 mM MES, 5 mM Tris and 400 mM sorbitol, pH 7.5 and an osmolality 650 mOsm. The pipettes were filled with a solution containing 100 mM KCl, 5 mM MES, 5 mM Tris and pH 6.0, which was adjusted to an osmolality of 580 mOsm with sorbitol. We decided to use 0.5 mM EDTA in the bath solution to destroy the Ca^2+^-activating ion channels and ensure the exclusive registration of the FV current, which is in agreement with the experiments that were recently performed by Bonales-Alatorre et al. [32,33]. However, in contrast to those authors, we did not add EDTA to the pipette solution because of two facts—first, in physiological conditions, vacuolar Ca^2+^ is present at a high concentration and second, an abrupt increase of the inward FV current possibly reflects a sudden decrease of the free vacuolar Ca^2+^ when EGTA becomes the dominant buffer, as was mentioned recently by Bonales-Alatorre et al. [32].

All of the electrophysiological experiments were performed in the two patch-clamp configurations, a whole-vacuole and an excised cytosolic side-out patch [36] using an EPC-7 Plus amplifier (List-Medical-Electronic, Darmstadt, Germany). The current and voltage conventions were in accordance with Bertl et al. [37], e.g., the sign of the voltage refers to the cytosolic side and the positive (outward) currents represent an efflux of cations into a vacuole. The signal was filtered using a five-pole Bessel filter and was recorded on a hard disk at a sampling frequency of 1–100 kHz. The Bessel filter was an integral part of the EPC-7 Plus amplifier. The whole-vacuole FV currents in symmetrical 100 mM KCl or a ten-fold vacuole-to-cytosol KCl gradient were elicited via a series of voltage ranging from −140 to +140 mV in 20 mV steps. From a holding potential of −40 mV, where the activity of the FV channels is minimal [21,22], the electrical potential across the tonoplast was changed for 3 s. The effect of 0.1, 1 and 10 μM indole-3-acetic acid (IAA) on the FV channels was studied. The bath solution in the recording chamber was exchanged via the continuous perfusion of the measuring chamber using an SP200 infusion pump (World Precision Instruments, USA). All of the experiments were performed at room temperature (22 ± 1 °C).

The patch pipettes were pulled from borosilicate glass tubes (Kimax-51, Kimble Products, Toledo, OH, USA) using a two-stage pipette puller (model L/M-3-PA, List Medical, Darmstadt, Germany), fire-polished using a CPZ 101 microforge (List Medical, Germany) and coated with Sylgard (Dow Corning, Midland, MI, USA). The patch electrode resistance (for the electrodes that had been filled with the pipette solution) was 2–4 MΩ; the gigaseal resistance was in the range of 5–20 GΩ. The voltage pulse within a range of 300–900 mV combined with gentle suction permitted access to the vacuole interior.

The experimental data were stored and elaborated using Patch-Master, Fit-Master software (HEKA Electronic, Lambrecht, Germany) and Dell Statistica (data analysis software system), version 13 (Dell, Texas, TX, USA). The details of the experiments and an analysis of the results were performed in accordance with our earlier papers [25,38]. The open probability was calculated (using FitMaster software) as the total opening time normalized to the total recording time and the number of active channels in a patch (see also [26,39]).

The free Ca^2+^ concentration of the bath solution was calculated using the Ca-EGTA Calculator v1.2 program (University of California, Davis, CA, USA). Details about the composition of the solutions are given in the figure legends.

### Statistical Analysis

The data were analyzed using Dell Statistica (data analysis software system), version 13 (Dell, Texas, TX, USA). The normal distribution was evaluated by a Shapiro–Wilk test (*p* > 0.05). If the normal distribution was confirmed the statistical differences were analyzed using independent samples *t*-test (*p* < 0.05) or paired *t*-test (*p* < 0.05).

If data failed the normal distribution then non-parametric Mann–Whitney *U*-test (asymptotic significance two-tailed, *p* < 0.05) was used.

## 5. Conclusions

In the plant vacuolar membrane, fast activating currents, which are carried by the FV channels, dominate the electrical characteristics at physiologically cytoplasmic free Ca^2+^ concentrations (<1 µM) [18]. Under low cytosolic free Ca^2+^ conditions, the fast activating vacuolar (FV) channels represent the main pathway for the K^+^ uptake and release across the vacuolar membrane [21,22,30]. Using the patch-clamp technique in whole-vacuole and cytosolic side-out configurations, we characterized the electrical properties of the FV channels in the absence and presence of IAA. Under the experimental conditions that were used here, the fast-activating vacuolar (FV) channels predominantly had the function of an outward-rectifying K^+^ channel, which, similar to the slow-activating vacuolar (SV) channels, enables the K^+^ efflux from the cytoplasm into the vacuole. At symmetrical 100 mM KCl, auxin stimulated an outward current of the fast-activating vacuolar K^+^ channels, which mediate the K^+^ efflux from the cytoplasm into the vacuole, but it did not change the K^+^ efflux from the vacuole into the cytoplasm. However, in conditions in which a ten-fold gradient of KCl was imposed on the vacuolar membrane, auxin diminished the outward current. Luminal auxin reduced both the outward and inward currents compared to the control. The single channels, which were recorded in symmetrical KCl, demonstrated that cytosolic auxin changed the open probability of FV channels at positive voltages to a moderate extent, while it significantly increased the amplitude of the single channel outward currents as well as the number of open channels. At the positive voltages, auxin did not change the unitary conductance of the single channels. The results presented here suggest that auxin regulates the activity of the fast-activating vacuolar (FV) channels, thereby causing changes of the K^+^ fluxes across the vacuolar membrane.

In the future we would like to investigate the effects of abscisic acid (ABA, a negative regulatory hormone), alone and in combination with IAA on activity of FV channels.

## Abbreviations

IAA	indole-3-acetic acid
FV	fast-activating vacuolar channel
SV	slow-activating vacuolar channel
